# Preparation and Characterization of Temperature-Triggered Microcapsules Fabricated via Low-Temperature Shear Method

**DOI:** 10.3390/ma19091799

**Published:** 2026-04-28

**Authors:** Zhitian Xie, He Wang, Wei Song, Chentao Xu, Shicheng Liu, Xiaokai Niu, Meng Qi

**Affiliations:** Beijing Municipal Engineering Research Institute, Beijing 100037, China; mlfgnr@163.com (Z.X.); wh3100206660@163.com (H.W.);

**Keywords:** microcapsules, sodium silicate, hydroxypropyl methylcellulose, temperature-triggered, low-temperature shear

## Abstract

**Highlights:**

- A novel composite core design strategy is proposed for the encapsulation of highly alkaline sodium silicate.
- A temperature-responsive microcapsule system is fabricated via a one-step low-temperature shear method.
- HPMC serves triple functions: chemical buffer, rheology modifier, and built-in temperature trigger.
- The microcapsules exhibit favorable short-term alkali resistance and distinct temperature-triggered release behavior.
- This work provides a new methodological approach for the design of stimuli-responsive microcapsules for cement-based grouting materials.

**Abstract:**

Emergency leakage repair in subway shield tunnels requires a technique to encapsulate highly reactive sodium silicate that is simple and field-deployable, yet no mature solution currently exists. The challenge lies in sodium silicate’s strong alkalinity and high osmotic pressure, both of which corrode most shell materials. This study proposes a “composite core” concept—functionally re-engineering the core rather than relying on complex shell chemistries. Using hydroxypropyl methylcellulose (HPMC) as the key material, temperature-triggered microcapsules with a nano-silica shell and sodium silicate–HPMC core were fabricated via low-temperature shear. Low temperature (10–15 °C) is critical: it suppresses side reactions and tunes viscosity to 2000–5000 cP, facilitating shear dispersion. The resulting microcapsules exhibit well-defined morphology with a dense shell. Temperature response tests reveal distinct release onset at ~30 °C (HPMC’s LCST): HPMC chain collapse generates internal stress that ruptures the shell, driving progressive sodium silicate release. Alkaline resistance tests confirm that intact microcapsules remain stable in high-pH environments (pH ≈ 13.2) for 30 min. This work validates the “composite core” concept and provides a simple, field-operable route to fabricate temperature-triggered microcapsules for emergency repair applications.

## 1. Introduction

Microencapsulation technology, which enables the encapsulation of substances within a protective shell and their controlled release under specific environmental stimuli, has emerged as a promising direction for the design of novel functional materials. This technology has been widely applied across diverse fields, from drug delivery to self-healing polymers [1,2,3,4,5,6]. The core value of microencapsulation lies in its ability to achieve spatiotemporal delivery of functional agents, thereby endowing materials with dynamic responsive capabilities.

However, when this technology transitions from laboratory settings to complex and harsh engineering applications such as civil engineering and chemical industries, its stability and controlled release performance often face severe practical challenges. In practical applications, microcapsules are frequently subjected to constraints imposed by temperature, pH, pressure, and other conditions. Under such conditions, microcapsules must not only protect their core materials but also possess shells capable of withstanding prolonged exposure to harsh environments. Taking cement-based materials as an example, cement paste maintains a highly alkaline environment for extended periods and contains high concentrations of diverse multivalent ions such as Ca^2+^, Al^3+^, and Fe^3+^ [7,8,9]. Under such aggressive conditions, conventional microcapsules are prone to failure due to shell dissolution, osmotic pressure imbalance, or ionic attack, making direct application difficult. Therefore, developing a robust encapsulation system capable of adapting to harsh environments has become a critical challenge for advancing the engineering application of microencapsulation technology.

In scenarios such as emergency leakage remediation in subway shield tunnels, cement-based materials must maintain good fluidity during initial placement and then undergo rapid setting once the grout reaches the target location [10]. Sodium silicate is widely used as an efficient setting accelerator due to its rapid reaction with Ca(OH)_2_, a product of cement hydration, to form C-S-H gel [11]. However, the high reactivity of sodium silicate leads to issues such as rapid paste setting, localized alkalinity surge, and uncontrolled hydration heat when added directly. Microencapsulation effectively isolates sodium silicate from the cement paste. This enables on-demand release during later grouting stages, thereby improving material performance and utilization efficiency [12]. Nevertheless, the inherent strong alkalinity and high osmotic pressure of sodium silicate solutions readily corrode most shell materials and induce shell rupture due to substantial osmotic pressure differences. From an engineering practice perspective, the challenges are even more pronounced. Existing research predominantly employs chemical methods such as interfacial polymerization and in situ polymerization for core encapsulation [13]. These processes are typically complex, require stringent conditions, and the long-term stability and triggering reliability of the resulting microcapsules in real cement environments remain unclear. More critically, in on-site scenarios like tunnel leakage emergency repair, the construction window is extremely short and working conditions are rudimentary, rendering complex chemical reactions or precise control unfeasible [14]. Consequently, developing a physical encapsulation method characterized by simple processing, low equipment requirements, and suitability for on-site engineering conditions is a core requirement for transitioning microencapsulation technology from the laboratory to engineering applications.

To address this challenge, this study proposes a novel strategy that integrates material innovation with simple physical processing. We hypothesize that the key to successful preparation lies not in constructing complex shell chemical structures but in the functional reconstruction of the core material itself. Accordingly, this study innovatively proposes the “composite core system” design concept, using hydroxypropyl methylcellulose (HPMC) as the core functional material to construct a sodium silicate–HPMC composite core system. This system provides chemical buffering and physical stability functions during preparation and storage, and can actively respond to temperature signals to trigger release during application. To avoid the engineering adaptability problems caused by cumbersome synthesis, this study uses a simple and efficient mechanical shearing technique to construct the shell layer. The key innovation of this study is that hydroxypropyl methylcellulose and sodium silicate form a composite core. During the shearing process, HPMC can self-assemble at the interface between the core material and hydrophobic nano-silica particles to form an intelligent responsive core system with phase change function [15,16]. The distinct LCST characteristic of HPMC at 30 °C enables the molecular chains to actively shrink and generate internal stress under temperature stimulation, ultimately achieving targeted release of the core material at the set temperature conditions [17,18].

On this basis, based on the classic Pickering emulsification mechanism, this study developed a simple low-temperature shearing method to prepare temperature-triggered microcapsules with hydrophobic nano-silica as the shell layer and sodium silicate–HPMC composite solution as the core material [19]. This paper aims to systematically validate and elucidate this material design philosophy of achieving multifunctional integration through simple processing. It systematically elaborates on the design principles, preparation method, and performance characterization of the temperature-responsive microcapsules. This research not only provides a practical new material for the functionalization of cement-based materials but also aims to offer a new methodological approach for encapsulation technologies intended for harsh environments.

## 2. Microcapsule Design Concept

### 2.1. Fundamental Material Selection

Based on the material properties of nano-silica and its high compatibility with the target process, this study selects hydrophobic nano-silica as the shell material. Its core advantage lies in its ability to utilize the Pickering emulsification mechanism: under mechanical shear, nano-silica particles can irreversibly adsorb onto the interface of core droplets, densely arrange at the interface, and form a dense inorganic nanoparticle shell layer through interparticle van der Waals forces and subsequent dehydration condensation of silanol groups. This structure is essentially a physical barrier with extremely low porosity, offering superior barrier performance against water and ions compared to most polymer films. Furthermore, the thermal stability and chemical inertness of nano-silica ensure the fundamental reliability of the shell in subsequent environments [20,21,22,23,24,25]. These characteristics collectively establish it as an ideal framework for constructing heterogeneous, multifunctional core–shell structures. The preparation method centered on mechanical shear avoids complex chemical reactions and aligns with the principle of simplicity and scalability.

### 2.2. Fundamental Encapsulation Challenges

Preliminary experiments revealed that while a sodium silicate solution could be transiently encapsulated within nano-silica via high-speed mechanical shear, the resulting microcapsules were extremely unstable, rapidly disintegrating, coalescing, and ultimately forming a milky white viscous solution. This outcome was not a preparation process failure but stemmed from fundamental thermodynamic and kinetic incompatibilities.

Firstly, the alkaline environment of the sodium silicate solution directly attacks nano-silica. The high alkalinity promotes ionization and dissolution of silanol groups (Si-OH) on the nano-silica surface. This destabilizes the nanoparticles and induces electrostatic repulsion between them. As a result, the adsorbed particle layer is disrupted, and the shell material cannot form a stable barrier [26].

Secondly, the high ionic strength of sodium silicate creates a substantial osmotic pressure difference across the shell. Any microscopic defects in the shell become channels for rapid water ingress, causing droplet swelling and shell rupture. Simultaneously, the spontaneous condensation polymerization of sodium silicate at ambient temperature drastically alters the core’s viscosity and volume. The internal stress generated by this process further compromises structural integrity [27,28].

Under the coupled influence of these factors, even if microcapsules form transiently during encapsulation, they rapidly disintegrate. More critically, even if the first two challenges were temporarily resolved through complex means, such a static microcapsule would lack a triggering mechanism within the cement paste environment, rendering it incapable of on-demand setting acceleration.

### 2.3. Proposed Solution

To address the aforementioned challenges, this study proposes a composite core system hypothesis: under complex conditions, the key to successfully encapsulating sodium silicate lies not in constructing an elaborate shell structure but in reconstructing the core material itself, endowing it with the ability to autonomously regulate its properties during both the encapsulation and triggering phases. Realizing this concept requires the core material to simultaneously satisfy three principles:

1. Chemical Buffering Principle: The chemical corrosion of silica by sodium silicate originates from the irreversible hydroxyl attack and siloxane bond cleavage in a strongly alkaline environment, with the dissolution rate significantly exceeding the interfacial assembly rate. To interrupt this process, the system must establish a dynamic, reversible shielding network within the core to temporarily sequester free OH^−^ and silicate ions, slowing down corrosion of the shell material.
2. Physical Regulation Principle: The low viscosity and high osmotic pressure of the sodium silicate solution make it difficult to form and maintain stable micro-droplets under shear. Therefore, the material must possess the capability to autonomously regulate its macroscopic rheological behavior, transforming the Newtonian fluid of sodium silicate into a viscoelastic liquid exhibiting yield stress and pronounced shear-thinning behavior under shear conditions.
3. Phase-Change Triggering Principle: The ultimate goal of encapsulation is on-demand release, not static containment. Hence, the system must internally possess a state switch responsive to external stimuli. Upon receiving a triggering signal, this system should induce a strong, self-initiated change.

Based on these three rigorous theoretical principles, this study selects hydroxypropyl methylcellulose (HPMC) as the core functional material.

First, based on experimental phenomena and classic polymer solution theory, we propose the following scientific hypothesis for the chemical buffering mechanism of hydroxypropyl methylcellulose (HPMC): HPMC can construct a reversible molecular buffer layer in strongly alkaline sodium silicate solution. The molecular backbone of HPMC is cellulose, with hydrophilic hydroxypropyl groups regularly distributed on the chain. These groups are rich in ether bonds and hydroxyl groups. In strongly alkaline sodium silicate solution, these groups act as excellent electron donors. They can engage in ion–dipole and hydrogen-bonding interactions with hydrated sodium ions (Na(H_2_O)_n_^+^) and silicate ions (SiO_3_^2−^). These weak interactions may promote the formation of a dynamic, spatially restricted network structure between HPMC molecular chains and silicate ions. This network is not uniformly dispersed but locally enriched around the polymer chains, thus temporarily binding part of the highly active ions at the microscale and effectively inhibiting their diffusion [16].

This hypothesis is supported by the following indirect experimental evidence:

1. The addition of HPMC significantly inhibits the dissolution of nano-silica in sodium silicate solution, which cannot be explained by simple physical mixing;
2. The pH stability test shows that intact microcapsules exhibit no significant pH change in water for 24 h, indicating effective isolation of the strongly alkaline core material;
3. The alkaline resistance test confirms that microcapsules prepared with HPMC remain stable in pH = 13.2 solution for 30 min, while microcapsules without HPMC disintegrate immediately.

Second, HPMC imparts shear-activated rheological behavior. HPMC is a typical water-soluble semi-rigid polymer. When its concentration exceeds the critical overlap concentration, molecular chains form a transient physical cross-linked network through entanglement and hydrogen bonding, endowing the system with characteristic viscoelasticity. Its rheological behavior precisely aligns with the physical regulation requirement: at rest or under low shear rates, the network remains intact, exhibiting high viscosity; under high-speed shear, entanglements are pulled apart, and the hydrogen bond network is directionally disrupted, leading to a sharp viscosity decrease, thereby facilitating the dispersion of the core material into micron-sized droplets. Upon cessation of shear, the network structure recovers through segmental thermal motion. This dynamic rheological property is the prerequisite fluid mechanics condition for successful physical encapsulation [29].

Third, HPMC possesses the capability for trigger-induced rupture. HPMC exhibits a distinct thermoresponsive phase transition, specifically Lower Critical Solution Temperature (LCST) behavior, originating from the balance between hydrophilic and hydrophobic groups along its molecular chain. Below the LCST, the molecular chains are fully hydrated and extended. When the temperature rises above the LCST, hydrophobic interactions dominate, causing chain dehydration and cooperative coiling. This phase transition not only fulfills the requirement for external signal responsiveness but also satisfies two critical aspects of Principle 3: (1) Chemical shielding removal: Chain coiling disrupts the hydrogen bonds and ion-dipole interactions maintaining the localized complexation network, leading to the instantaneous release of sequestered active ions. (2) Internal stress generation: Within the confined space of the microcapsule, the global coiling of polymer chains translates into an isotropic contractile stress on the inner wall of the shell. According to polymer gel theory, the contractile stress generated by such a phase transition is substantial, sufficient to initiate microcracks in the nano-silica shell and promote their propagation [15,18].

In summary, HPMC simultaneously satisfies the three theoretical requirements of reversible chemical buffering, dynamic rheology, and phase-change dissociation. Other materials, such as non-ionic surfactants, lack sufficient thickening capacity and phase-transition stress; common thickeners like sodium polyacrylate lack environmental responsiveness; and simple thermoresponsive polymers like poly (N-isopropylacrylamide) may fail in strongly alkaline, high-salt environments. None can simultaneously meet all the stipulated principles. HPMC emerges as a particularly suitable candidate derived from the current theoretical framework. This constitutes the central scientific hypothesis of our entire study, and all subsequent experiments are designed to validate this hypothesis and its derived predictions [17].

### 2.4. Interfacial Interaction and Encapsulation Mechanism Analysis

HPMC molecules have a clear amphiphilic structure: the main chain and hydroxypropyl side chains provide hydrophilicity, while the hydrocarbon parts of methoxy and hydroxypropyl groups exhibit hydrophobicity. In solution, the hydrophilic segments of HPMC molecules extend into the aqueous phase, forming a dynamic composite core system through hydrogen bonding with silicate ions, and improving the viscoelasticity of the solution. The shear force first tears the composite solution into discrete droplets. We further propose that due to the amphiphilicity of free HPMC molecules in the solution, they will preferentially adsorb on the newly formed droplet-nano-silica powder interface during the shearing process. Their hydrophilic parts are anchored in the aqueous phase rich in sodium silicate, while the hydrophobic parts (hydrocarbon segments of methoxy and hydroxypropyl groups) naturally face the hydrophobic nano-silica particles.

We speculate that the HPMC molecular layer plays a dual role immediately after the contact between droplets and silica particles: on one hand, it isolates the direct contact between strongly alkaline sodium silicate and the surface of nano-silica; on the other hand, it anchors the silica particles on the droplet surface through hydrophobic interactions. Under continuous shearing, the anchored nano-silica particles collide and rearrange around the droplets, and finally complete dense self-assembly on the droplet surface through interparticle interactions to form a complete shell layer [15,30].

## 3. Experimental Design

### 3.1. Experimental Materials and Instruments

Materials: Liquid sodium silicate (water glass), 50°Bé, produced by Tianjin Zhonglian Chemical Reagent Co., Ltd. (Tianjin, China); Hydrophobic nano-silica, produced by Aladdin Reagents (Shanghai, China); Hydroxypropyl methylcellulose (HPMC), viscosity: 15 mPa·s, produced by Aladdin Reagents; Rhodamine 6G dye, produced by Tianjin Huasheng Chemical Reagent Co., Ltd. (Tianjin, China).

Instruments: NDJ-5S Digital Rotational Viscometer (Lichen Instrument Technology Co., Ltd., Shaoxing, China); PHS-3C pH Meter (Hangzhou Qiwei Instrument Co., Ltd., Hangzhou, China); Thermostatic Heating Magnetic Stirrer with Oil/Water Bath (Shanghai Lichen Bangxi Instrument Technology Co., Ltd., Shanghai, China); Overhead Electric Stirrer (Shanghai Lichen Bangxi Instrument Technology Co., Ltd., Shanghai, China); Constant Temperature Magnetic Stirrer (Changzhou Surui Instrument Co., Ltd., Changzhou, China).

### 3.2. Core Material Ratio Experiment

To verify the necessity of a low-temperature environment for forming a processable core precursor and precisely define the successful encapsulation processing window, this study designed a systematic temperature gradient experiment. This experiment aimed to investigate the influence of preparation temperature on the rheological properties of the sodium silicate–HPMC composite core system, thereby providing clear parameter guidance for the subsequent shear encapsulation process.

To elucidate the regulatory effects of temperature and HPMC dosage on the behavior of the composite system and to determine the material ratios for microcapsule preparation, a two-factor, multi-level experiment was designed:

Factor 1: Preparation Temperature (T): Set at three levels: 25 °C, 15 °C and 5 °C.

Factor 2: HPMC Dosage (C): Set at five levels relative to the mass of sodium silicate solution: 0%, 1%, 2%, 4%, and 8%.

For each temperature and dosage combination, the experiment was performed in the following sequence:

Core Material Preparation: A total of 100 g of sodium silicate solution was placed in a thermostatically controlled ice-water bath and precisely adjusted to the target temperature. Under continuous stirring at 800 rpm, the corresponding mass of HPMC powder was slowly added. Stirring continued until the material was uniformly mixed, achieving a visually homogeneous state.

Viscosity measurement. An NDJ-5S digital rotational viscometer (No. 2 rotor, 12 rpm) was used to measure the steady-state viscosity of the system under the corresponding constant temperature conditions. Each sample was thermostatically equilibrated for 3 min before measurement. Each sample was measured in parallel three times, and the average value was taken.

The orthogonal experimental design table for this section is shown in Table 1 below.

**Figure 1:**
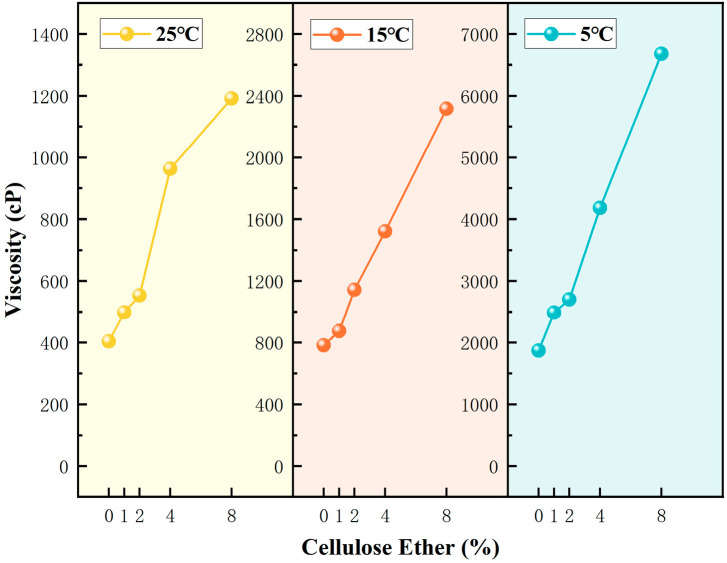
Variation in viscosity of sodium silicate–cellulose ether solutions at different temperatures and mass ratios.

The experimental results are shown in Figure 1.

At the same HPMC dosage, the static viscosity of the system showed a significant upward trend with decreasing temperature. This change law originates from the inherent viscosity–temperature dependence of cellulose ether aqueous solutions: under low temperature conditions, molecular thermal motion is weakened, and the hydrogen bonding interactions between HPMC molecular chains, water molecules and silicate ions are significantly enhanced. The transient physical crosslinking network formed between molecular chains is more stable, thus the static viscosity of the system is greatly increased; while increasing temperature will destroy the hydrogen bonding network and untangle the molecular chains, leading to a decrease in system viscosity. This rheological behavior is completely consistent with the inherent characteristics of HPMC aqueous solutions, and is also one of the core reasons why this study chose a low-temperature environment for microcapsule preparation.

At a fixed temperature, viscosity increased with increasing HPMC dosage. Notably, at 25 °C, even with increased HPMC dosage, the viscosity increase was very limited, indicating that the hydrogen bond network is difficult to establish stably at elevated temperatures, and side reactions may have compromised the thickening efficacy of HPMC [11,16].

Based on preliminary preparation exploration, a viscosity range of 2000–5000 cP is favorable for forming stable, uniform droplets via mechanical shear. If the viscosity is too high, the internal intermolecular forces are strong, hindering molecular motion and making effective dispersion difficult. Conversely, if the viscosity is too low, the fluidity is excessive, shear energy dissipates rapidly, leading to unstable droplet formation, liquid escaping from shear gaps, or coalescence, hindering effective shell coverage. To ensure the effectiveness and repeatability of the encapsulation process itself, we first rationally optimized the core-to-shell mass ratio. Based on preliminary experiments, when the mass ratio of sodium silicate solution to HPMC was 25:1 and the ratio to nano-silica was 10:1, the system macroscopically achieved the highest encapsulation efficiency and stable dispersion state. Consequently, all subsequent preparation experiments were conducted using this optimized formulation.

### 3.3. Low-Temperature Shear Preparation of Microcapsules

At the same HPMC dosage, the system viscosity increased significantly with decreasing temperature. This trend originates from the inherent viscosity–temperature dependence of cellulose ether aqueous solutions: low temperature weakens molecular thermal motion and enhances hydrogen bonding interactions between HPMC chains, water molecules, and silicate ions, thereby stabilizing the transient physical crosslinking network and substantially increasing the static viscosity of the system.

At a fixed temperature, viscosity increased with increasing HPMC dosage. Notably, at 25 °C, even with increased HPMC dosage, the viscosity increase was very limited, indicating that the hydrogen bond network is difficult to establish stably at elevated temperatures, and side reactions may have compromised the thickening efficacy of HPMC [24,31,32].

The specific steps for microcapsule preparation are as follows:

(1) Preparation of Sodium Silicate–HPMC Aqueous Phase

Place 50 g of sodium silicate solution in a thermostatically controlled ice-water bath, precisely controlling the bath temperature according to the five experimental condition groups: 5 ± 1 °C, 10 ± 1 °C, 15 ± 1 °C, 20 ± 1 °C, 25 ± 1 °C. Immerse a thermometer into the sodium silicate solution to monitor its temperature, maintaining it within the target fluctuation range. Slowly and uniformly add 2 g of HPMC to the solution, stirring continuously at 800 rpm using a mechanical stirrer until the system appears milky white and homogeneous with no visible particle agglomerates.

(2) Shear-Induced Interfacial Stabilization

Place 5 g of nano-silica in a beaker, preferably one with a smaller opening and diameter. Adjust the stirring blade depth to approximately the lower 1/3 of the powder layer, ensuring the nano-silica covers the stirring blade but preventing contact between the blade and the beaker bottom or walls. Set the stirring speed to 1800 rpm. Under high-speed shear at 1800 rpm, slowly and uniformly pour the prepared sodium silicate–HPMC solution into the nano-silica powder. Continue shearing for 5 min to allow the nanoparticles to form a dense adsorbed layer on the droplet surface. Finally, pour out the solid material from the beaker and sieve it sequentially through two sieves: first through a coarse sieve with an aperture of approximately 0.8 mm to remove large agglomerates, and then through a fine sieve with an aperture of approximately 0.1 mm to eliminate unencapsulated fine powder. The material retained on the fine sieve was collected as the final microcapsule product.

(3) Post-Treatment

At ambient temperature, sodium silicate and HPMC can react, potentially leading to the internal degradation of the nano-silica microcapsules. Therefore, post-treatment of the microcapsules is necessary. They can be stored temporarily in a low-temperature environment (<4 °C).

It should be noted that “low temperature”, in this study, is an engineering definition relative to the typical ambient temperature (20–25 °C) encountered at subway tunnel construction sites, rather than an absolute physical low temperature. As shown in Table 2, all experimental groups maintained the same core-to-shell mass ratio, with the only variable being the equilibrium temperature of the core solution prior to shear encapsulation. This series of experiments was designed to intuitively validate the critical temperature threshold required for successful microcapsule formation, as elaborated in the preceding sections.

### 3.4. Microcapsule Morphology Observation

A fluorescence microscope was used to observe the macroscopic morphology of the successfully prepared microcapsules, assessing their geometric integrity and particle size distribution. The particle size distribution and sphericity of microcapsules are core indicators for evaluating the stability of the encapsulation process. Furthermore, to analyze the microscopic morphology and structural details of the microcapsules, SEM was employed to observe the microscopic appearance of the microcapsules.

### 3.5. Microcapsule Temperature Response Test

To verify the temperature response characteristics endowed to the microcapsules by HPMC acting as an internal trigger, this study designed a gradient heating release experiment. The experiment used microcapsules prepared at 15 °C as the subject. By monitoring the pH change in the suspension during heating, the release behavior of the core material (sodium silicate) was quantitatively characterized.

The design of the temperature response experiment was based on the following theoretical considerations:

Trigger Threshold Verification: According to the preceding discussion, the LCST phase transition of HPMC (approx. 30–35 °C) should act as the “switch” for release behavior. The inflection point of pH change corresponds to this phase transition temperature.

Release Kinetics Characterization: The pH change curve during heating can be converted into a cumulative release rate curve, facilitating a preliminary analysis of the release mechanism.

Shell Integrity Verification: A comparative experiment was set up between microcapsules (Group D1) and dried microcapsules (Group D2). The drying process promotes dehydration condensation between silanol groups on the nano-silica particles within the shell, enhancing its mechanical strength. If both groups exhibit similar triggering behavior, it would demonstrate that: (1) Drying did not damage the microcapsule structure; (2) The triggering mechanism originates from the intrinsic phase transition of the core material HPMC, not from accidental shell damage during drying. Drying conditions: 65 °C for 8 h.

To quantify the temperature sensitivity of the microcapsules and demonstrate the morphological integrity of the microcapsule surface, two sets of parallel experiments were designed. All experiments used microcapsules prepared at 15 °C. The core variable was whether the microcapsules underwent drying and solidification, and their dosage. The procedure for the microcapsule temperature response experiment was as follows:

Sample Pretreatment. A measured quantity of water was placed in a constant temperature reactor and its pH was measured. The microcapsule sample was added, stirred magnetically at 300 rpm for 5 min, and pH changes were monitored in real-time using a precision pH meter. Results showed a pH fluctuation range of ≤0.1 during stirring, indicating no residual alkaline substances on the microcapsule surface.

Temperature Gradient Release Experiment. The liquid was slowly heated using a constant temperature magnetic stirrer, and the liquid temperature was monitored with a thermometer. At 30, 35, 40, 45, and 50 °C, 5 mL aliquots of the suspension were withdrawn, immediately replacing the sampled volume with an equal amount of water. The withdrawn suspension was cooled to room temperature, and its pH was measured and recorded.

As shown in Table 3, Group D1-X used freshly prepared, undried microcapsules to simulate triggering behavior upon direct application after preparation. Group D2-X used dried microcapsules to demonstrate that the microcapsule surface morphology remained intact during preparation and was not damaged.

### 3.6. Microcapsule Alkaline Environment Durability Test

During cement hydration, the pH of the pore solution in the matrix increases significantly, creating a highly alkaline environment. Rapid degradation of the shell in this high-alkali environment would cause premature leakage of the core material before reaching the triggering temperature, rendering the “temperature trigger” design meaningless. To verify the stability of the microcapsules under such combined conditions and avoid premature shell degradation due to pH increase, this study designed an alkaline resistance experiment based on in situ fluorescence observation to evaluate the short-term stability of microcapsules in a high-alkali environment.

Given the requirements of the on-site tunnel repair window, the initial setting time of the cement matrix is controlled to approximately 30 min, and the influence of hydration product deposition on microcapsule compression is not considered [1,33]. Therefore, the duration of the pH stability experiment was controlled within 30–45 min based on practical requirements.

In this study, the response intensity of the microcapsule core material under conventional fluorescence excitation was limited, resulting in insignificant differences in fluorescence intensity before and after rupture. To obtain accurate and reliable rupture kinetics data, this study employed a direct counting method based on morphological observation. The effectiveness of this method relies on observing microcapsules before and after rupture. During the experiment, microcapsules in each sample field were observed periodically and continuously under the microscope, and statistics were recorded. The rupture rate was quantified by calculating the proportion of ruptured microcapsules relative to the total number. Each experiment was performed three times to ensure reliability. The procedure for the microcapsule alkaline environment durability experiment was as follows:

1. Dye (1% by mass of sodium silicate) was dissolved in the sodium silicate solution and stirred until completely dissolved, forming a sodium silicate-dye mixed solution.
2. Microcapsules were prepared according to the preparation method in a low-temperature environment.
3. A small amount of dye-labeled microcapsules was taken and uniformly dispersed in the central area of a glass slide. Subsequently, one drop of sodium silicate solution (50°Bé, pH = 13.2) was precisely applied to completely cover the microcapsule population.
4. The prepared slide was placed on the fluorescence microscope stage. A systematic scan was performed to select a typical field containing at least 20 intact microcapsules. Dynamic in situ observation of this area was conducted continuously for 30–40 min, capturing high-resolution images every 5 min, with a focus on recording changes in microcapsule morphological integrity.
5. Microcapsules in the field of view were counted, and the damage rate was calculated.

As shown in Table 4, this is the experimental grouping for the microcapsule alkaline resistance test, with the core variable being the preparation temperature.

## 4. Experimental Results and Analysis

The successful triggering and release of the microcapsule system in the complex cement-based environment requires meeting three fundamental requirements: structural integrity, stability, and triggering sensitivity. This section focuses on validating the inherent properties of the microcapsules themselves to ensure their usability in cement-based materials, systematically verifying whether the prepared microcapsules possess these three key qualifications as competent system components.

### 4.1. Microcapsule Morphology Analysis

**Figure 2:**
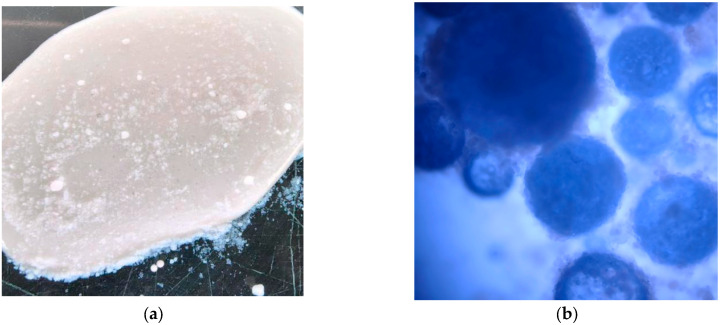

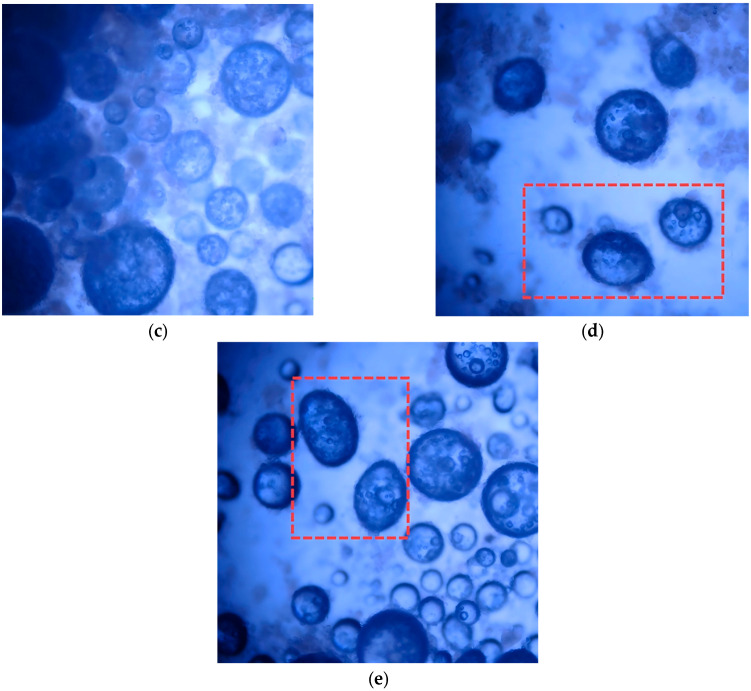
Fluorescence microscopy images of microcapsules prepared at different temperatures. (**a**) T1 Unsuccessful encapsulation mixture at 25 °C. (**b**) T2 Microcapsules successfully prepared at 20 °C. (**c**) T3 Microcapsules successfully prepared at 15 °C. (**d**) T4 Microcapsules successfully prepared at 10 °C. (**e**) T5 Microcapsules successfully prepared at 5 °C.

The primary prerequisite for achieving the controlled release of sodium silicate is its effective encapsulation within a dense physical barrier. Figure 2 shows the macroscopic morphology of microcapsules prepared by the mechanical shear method in water baths at 25, 20, 15, 10, and 5 °C, respectively.

At room temperature (25 °C), the system failed to form discrete microcapsules; instead, macroscopic phase separation with noticeable stratification and agglomeration of sodium silicate solution and nano-silica powder occurred (Figure 2a). This indicates that at room temperature, the rapid condensation of sodium silicate and the swift irreversible interaction between sodium silicate and HPMC resulted in a low-viscosity solution. This solution lost the dynamic complexation capability and thermal responsiveness of HPMC, failing to resist Ostwald ripening under mechanical shear to form discrete small droplets, let alone being encapsulated by nano-silica. Instead, the highly reactive sodium silicate underwent bulk reaction with a large amount of silica powder during shearing, forming gel-like agglomerates rather than dispersed microcapsules [31,33,34]. This result demonstrates that for this system, the conventional room-temperature preparation route is ineffective.

As the temperature decreased below 20 °C, discrete microcapsule particles began to appear, and the microcapsule morphology was roughly spherical with a wide size distribution, mainly ranging from 300 to 800 μm. This study defines 25 °C (room temperature) as an unsuitable high-temperature condition for preparation, and the core basis includes two aspects: viscosity regulation and side reaction inhibition. Among them, the self-condensation of sodium silicate and the degradation of HPMC in alkaline environment are typical chemical reactions, and their reaction rates and temperature relationships follow the Arrhenius equation, that is, the reaction rate decreases exponentially with decreasing temperature. Low temperature conditions effectively inhibit the above harmful side reactions, providing key thermodynamic driving force and sufficient time window for HPMC molecular chains and sodium silicate ions to construct a dynamic complex network through reversible hydrogen bonds and ion-dipole interactions; at the same time, low temperature adjusts the solution viscosity to an appropriate range, enabling mechanical shear force to overcome internal forces, form uniform droplets, and be coated by hydrophobic nano-silica particles through interfacial self-assembly.

In the temperature range of 10–15 °C, microcapsule particles with regular morphology and good sphericity were successfully obtained. The product exhibited a relatively concentrated particle size distribution. This indicates that within this temperature window, the dynamic hydrogen bond network formed by HPMC endowed the system with ideal pseudoplastic fluid behavior, enabling effective deformation and dispersion in the shear field and rapid recovery of high viscosity upon shear cessation to resist droplet coalescence, thus achieving a stable, controlled emulsification process.

When the temperature dropped below 10 °C, microcapsules were still successfully prepared, and their average diameter continued to decrease, stabilizing in the 200–500 μm range. However, non-spherical particles began to appear, some exhibiting ellipsoidal or spindle-like morphologies. This is because excessively low temperatures, while inhibiting reaction rates, led to excessively high solution viscosity and imbalanced interfacial tension. Droplets deformed by shear could not fully retract into spheres due to insufficient interfacial tension.

**Figure 3:**
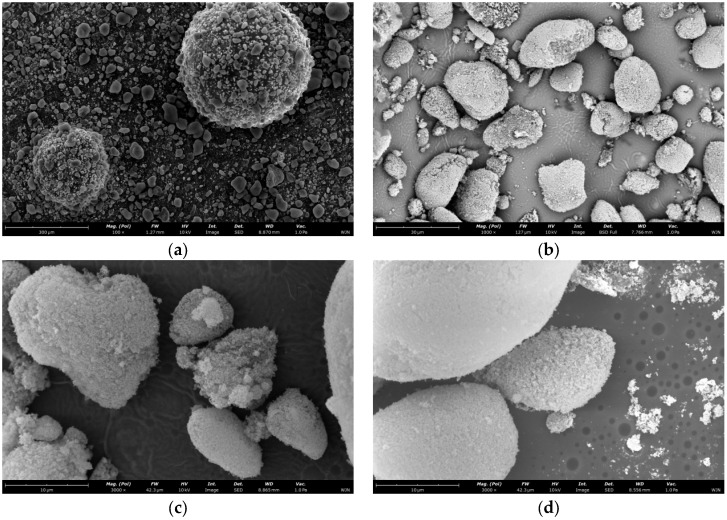
Scanning electron microscopy (SEM) images of the microcapsules (pre-rupture): (**a**) low-magnification panoramic view showing the overall size distribution, sphericity and dispersity of the microcapsules; (**b**) high-magnification surface morphology im-age (×1000); (**c**) high-magnification surface morphology im-age (×3000); (**d**) high-magnification surface morphology im-age (×3000).

To investigate the microstructure of the microcapsule shell, we observed the samples using SEM. As shown in Figure 3, All four SEM images are of microcapsule samples from the same batch fabricated at 15 °C, with identical instrumental parameters including accelerating voltage and working distance. Figure 3a is a low-magnification panoramic view showing the overall size distribution, sphericity and dispersity of the microcapsules. Figure 3b–d are progressively magnified surface morphology images, which clearly demonstrate the compactness, surface roughness and intact characteristics of the microcapsule shell layer without obvious pores or cracks., the dried and cured microcapsules have smooth, dense surfaces with no obvious defects. The microcapsule shell is formed by the self-assembly of nano-silica particles. This continuous and complete shell structure confirms that hydrophobic nano-silica particles form a continuous and tight packing layer on the surface of core droplets. It provides the necessary physical barrier to isolate the strongly alkaline core material from the external cement environment. This initially meets the basic requirements for encapsulation.

Combined with the classic capsule formation mechanism of Pickering emulsification and subsequent experimental results, the observed morphology strongly suggests that the microcapsules prepared in this study possess a well-defined core–shell architecture. The shell appears to consist of a densely packed layer of hydrophobic nano-silica particles, while the core is presumed to contain the sodium silicate–HPMC composite system. SEM characterization provides direct morphological evidence for the compactness and apparent lack of macroscopic defects in the shell layer. While direct cross-sectional imaging would provide definitive confirmation of the core–shell interface, the combination of indirect evidence presented herein is fully consistent with the formation of an intact core–shell structure.

### 4.2. Influence of Preparation Temperature on Microcapsule Particle Size Distribution

**Figure 4:**
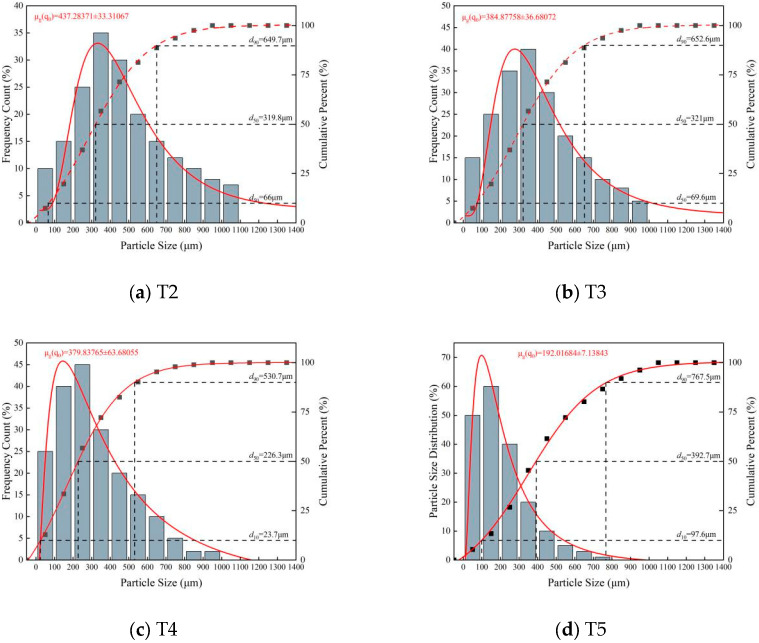
Particle size distribution of microcapsules prepared at different temperatures. Red line: Fitted particle size frequency distribution (with fitting equation). Black dots: Experimental cumulative particle size distribution. Red fitting curve: Fitted cumulative distribution.

The concentration and dispersion of the particle size distribution are not only core indicators for evaluating the stability of the microcapsule preparation process but also crucial clues for revealing the mechanisms of droplet formation and stabilization. To quantify the size characteristics of microcapsules prepared at different temperatures, systematic particle size statistics were performed on samples from each temperature gradient using ImageJ software (https://imagej.net/) based on fluorescence microscopy images. The results are shown in Figure 4. It can be clearly observed from Figure 4 that the preparation temperature has a decisive influence on the particle size distribution of the microcapsules, showing a distinct regular trend:

For the T2 group (20 °C), the particle size distribution was relatively broad, ranging from tens of micrometers to over 1000 μm. The fitted curve showed a main peak between 400 and 500 μm, with significant tailing. This proves that at this temperature, although sodium silicate condensation and HPMC side reactions were partially inhibited, they were not completely blocked, leading to unstable interfacial tension during emulsification, causing coalescence and Ostwald ripening of some droplets, forming abnormally large particles.

As the temperature decreased, the particle size distribution narrowed, the main peak of the fitted curve gradually shifted forward to 100–200 μm, and larger-sized microcapsule particles gradually decreased.

These distribution characteristics indicate that as the temperature decreases, low temperature effectively regulates the rheological behavior of the core system by inhibiting side reactions, forming uniform droplets in the shear field and effectively resisting coalescence. This provides an ideal environment for the subsequent self-assembly behavior of nano-silica particles, thus laying the foundation for successful encapsulation.

### 4.3. Microcapsule Temperature Response and Release Mechanism

**Figure 5:**
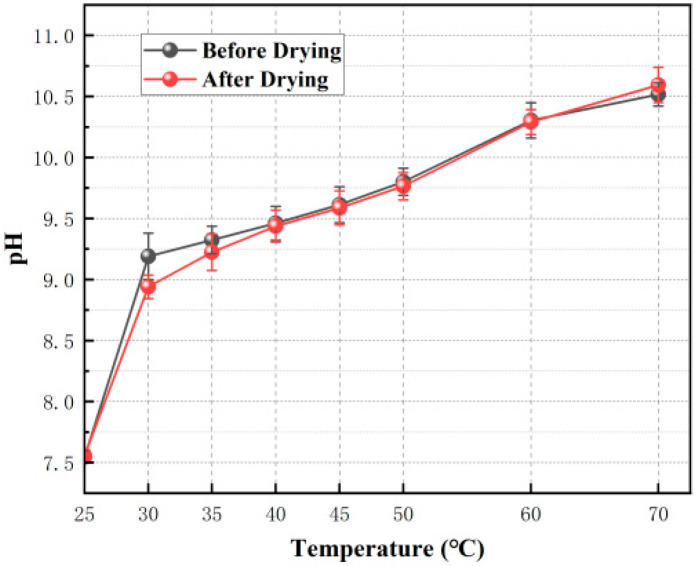
pH variation in dried and undried microcapsule suspensions at different temperatures.

Building on the confirmation of structural integrity, the temperature response characteristics of the microcapsules needed verification. A gradient heating release experiment was designed. The experiment used microcapsules prepared at 15 °C as the subject. By monitoring the pH change in the suspension during heating, the release behavior of the core material (sodium silicate) was quantitatively characterized. The experimental results are shown in Table 5 and Figure 5. The experiment demonstrated the following:

1. For an individual microcapsule, crack propagation is a gradual process; the core material slowly exudes through propagating microcracks.
2. Individual microcapsules exhibit variability in shell thickness and defect distribution, leading to dispersion in their actual rupture temperatures, causing the population release behavior to display temperature-dependent broadening.

**Table 5:** pH values recorded during the temperature-triggering test of microcapsules.

*Temperature*	*D 1-1*	*D 2-1*	*D 1-2*	*D 2-2*	*D 1-3*	*D 2-3*	*Mean*		*Std Dev*	
							*D 1*	*D 2*	*D 1*	*D 2*
25 °C (water)	7.5	7.6	7.5	7.5	7.6	7.57	7.5333	7.55667	0.05033	0.05132
25 °C (After adding Microcapsules)	7.5	7.6	7.5	7.6	7.62	7.6	7.54	7.6	0.06928	0
25 °C (After stirring)	7.54	7.6	7.52	7.6	7.65	7.6	7.57	7.6	0.07	0
30 °C	9.24	8.9	9.35	9.05	8.98	8.87	9.19	8.94	0.19	0.09644
35 °C	9.29	9.21	9.45	9.38	9.23	9.08	9.32333	9.22333	0.11372	0.15044
40 °C	9.4	9.45	9.62	9.56	9.36	9.3	9.46	9.43667	0.14	0.13051
45 °C	9.56	9.58	9.78	9.73	9.5	9.45	9.61333	9.58667	0.14742	0.14012
50 °C	9.7	9.72	9.92	9.89	9.78	9.68	9.8	9.76333	0.11136	0.1115
60 °C	10.3	10.27	10.45	10.4	10.16	10.2	10.30333	10.29	0.14503	0.10149
70 °C	10.52	10.65	10.61	10.7	10.42	10.43	10.51667	10.59333	0.09504	0.14364

In Figure 5, it can be seen that the error bar is longest at 30 °C and tends to decrease with increasing temperature. This reflects the differential rupture of shell materials before and after the temperature trigger: microcapsules with local defects and non-uniform thickness rupture first, while at higher temperatures, the synchronicity of shell rupture and core release improves.

For the release curves of samples before and after drying, the trends were highly consistent. This similarity demonstrates the following:

1. The drying process did not damage the structural integrity of the microcapsules; the core material was effectively encapsulated before heating.
2. The essence of the triggering mechanism, i.e., HPMC phase-transition-driven internal stress rupture, remained unchanged despite shell enhancement.

However, the pH jump amplitude during the triggering phase was slightly smaller for the dried samples. This indicates that the drying treatment strengthened the mechanical integrity of the shell to some extent, requiring higher internal stress accumulation for the same degree of rupture, manifesting as a slight “blunting” of the trigger threshold, but without altering the essential nature of the temperature response.

To verify the reliability of pH as an indicator for sodium silicate release, this study prepared sodium silicate standard solutions. The concentration gradient was 0–0.4 g/L, based on “Industrial Sodium Silicate GB/T4209-2008 [35]”. We measured the pH values of each solution at 25 °C using a pH meter. Then, we established a calibration curve between pH value and sodium silicate mass concentration:

Microcapsule core material: 50 g 50°Bé sodium silicate + 2 g HPMC.

Shell material: 5 g nano-silica.

Total solid content: 57 g.

Release experiment: 1 g microcapsules dispersed in 400 mL pure water.

According to the industry-common 80% encapsulation efficiency, the maximum concentration of sodium silicate in water is 0.2947 g/L after complete release from 1 g microcapsules. Therefore, we set the concentration range of the calibration curve to 0–0.4 g/L.

The corresponding relationship between sodium silicate concentration and pH at 25 °C is shown in Table 6.

Linear fitting of the data gives: pH = 10.508X + 7.4958. The linear correlation coefficient R^2^ = 0.995.

(1) $$Cumulative\;release\;rate\;Q_{t}=\frac{Actual\;concentration\;C_{t}}{Theoretical\;maximum\;concentration\;C_{max}}\times 100\%$$

Substituting the data from Table 5 gives the above results. The results are shown in Table 7.

Fitting the data in the above table gives the linear fitting equations and correlation coefficients for each kinetic model. The results are shown in Table 8.

The corresponding model fitting verification results are shown in Table 7 and Table 8. The fitting results show that the Ritger–Peppas model has the highest correlation coefficient (R^2^ = 0.992) among the four models. This means this model can most accurately describe the release behavior of the microcapsules. The release index n of this model is 0.76, which is in the range of 0.45–0.89. This indicates that the release mechanism of the microcapsules is non-Fickian anomalous diffusion. It is a composite release mechanism controlled by both shell rupture and Fickian diffusion. Shell rupture is the dominant factor.

Combined with experimental phenomena and fitting results, the temperature-sensitive release mechanism of microcapsules is analyzed as follows: When the system temperature exceeds the LCST of HPMC (~30 °C), the HPMC chains undergo dehydration and collapse. This phase transition generates isotropic contractile stress against the inner shell wall within the confined microcapsule volume. Once this internal stress surpasses the strength threshold of the nano-silica shell, microcracks initiate and propagate, releasing the core material. The core material sodium silicate is released through these microcracks. This is the dominant factor of the release behavior. Fickian diffusion of sodium silicate through the cracks is the secondary factor. This conclusion is highly consistent with the composite core system design hypothesis proposed in this study.

**Figure 6:**
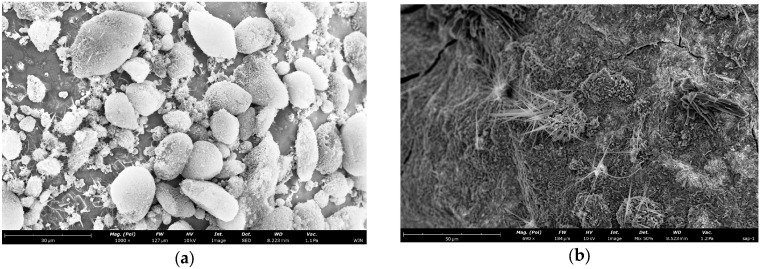

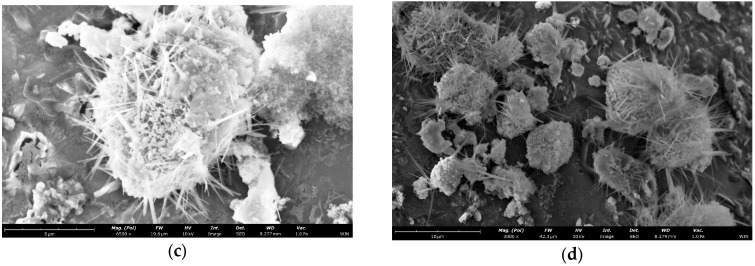
SEM images of microcapsules after temperature-triggered rupture (showing crystallized core material exudation). (**a**) Overall rupture morphology. (**b**) High-magnification surface morphology image. (**c**) High-magnification surface morphology image. (**d**) High-magnification surface morphology image.

**Figure 7:**
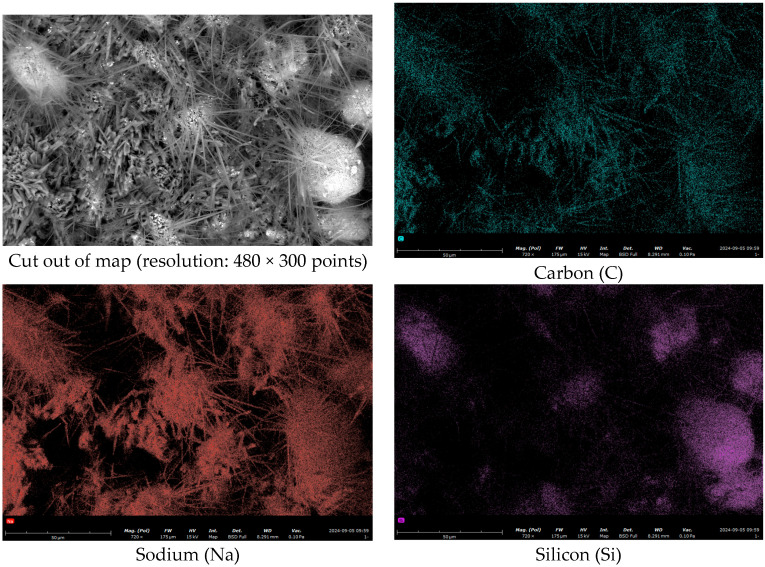
EDS elemental mapping of the precipitates on ruptured microcapsules.

To study the temperature-triggered release behavior, a group of freshly prepared microcapsules was immersed in a 25 °C water bath for 30 min to simulate the triggering process, then dried and observed by SEM. The resulting rupture morphology is shown in Figure 6. The ruptured microcapsules exhibited the following characteristic morphological features:

1. The shells of ruptured microcapsules largely maintained their original spherical contour, indicating that the cross-linked nano-silica shell possesses good structural toughness, sufficient to retain its skeletal integrity after releasing the core material. This also corroborates the aforementioned notion of gradual release rather than instantaneous rupture.
2. A large number of precipitated fibrous or acicular crystals were observed on the surface and edges of the ruptured microcapsules. As shown in Figure 7 and Table 9, EDS energy spectrum analysis showed obvious enrichment of carbon and sodium elements in these acicular crystals. These elements are highly consistent with the core material. The reason for the appearance of acicular crystals is as follows: Sodium silicate precipitates on the surface of microcapsules. It reacts with carbon dioxide in the air. Finally, visible acicular crystals are formed after water evaporation [32]. When the electron beam directly acts on these sodium and carbon-rich crystalline regions, a strong local signal enrichment appears. The EDS characterization results can directly confirm that the core material inside the microcapsules is successfully released to the outside after temperature triggering. This provides direct compositional evidence for the temperature-responsive release behavior of the microcapsules.

In summary, the temperature-triggered release experiments confirm our earlier hypothesis. The LCST phase transition of HPMC is the switch that starts the release process. The release process is gradual and depends on temperature. The mechanical strength of the shell can be adjusted by drying and other post-treatments. However, this does not change the temperature-responsive nature of the system. It should be emphasized that this study proposes a scientific hypothesis based on experimental results and polymer phase transition theory. Direct in situ observation of internal stress evolution and crack propagation in microcapsules during heating requires more advanced characterization techniques. These include in situ atomic force microscopy (AFM) and cryogenic transmission electron microscopy (cryo-TEM). This is also a key research direction in this field in the future.

### 4.4. Microcapsule Alkaline Resistance Test

**Figure 8:**
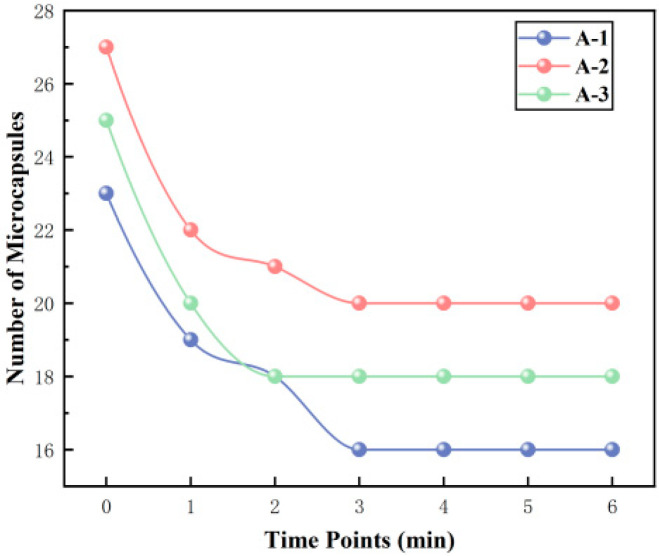
Number of microcapsules over time in an alkaline environment.

Cement matrices constitute a highly alkaline environment. To verify the alkali resistance of the microcapsules and prevent premature triggering due to the high alkalinity within the cement matrix, this study designed and conducted an in situ alkaline resistance experiment. Using 5 min intervals over six periods, the number of microcapsules within the field of view was systematically recorded and analyzed. Table 10 and Figure 8 show the changes in the number of microcapsules within the field of view during three experimental runs:

Using Equations (2) and (3) below, the cumulative rupture rate up to each time point and the rupture rate within each time interval can be calculated, respectively. The calculation results are presented in Table 10.

The cumulative rupture rate reflects the proportion of all ruptured microcapsules relative to the “initial total number” from the start of the experiment to a specific time t_i_. The formula is

(2) $$R_{cum,i}=\frac{\sum_{k=1}^{i}∆N_{broke,i}}{N_{0}}\times 100\%$$

$\sum_{k=1}^{i}∆N_{broke,i}$ is the total number of microcapsules ruptured from *t_0_* to *t_i_* (*i*.e., the cumulative number ruptured by time *t_i_*, with *i* = 1,2,3,4,5,6.

$N_{0}$ is the total number of microcapsules at the initial time (t_0_).

The interval rupture rate reflects the proportion of microcapsules ruptured within a specific time interval [*t_i−1_,t_i_*] relative to the total number at the beginning of that interval. The formula is

(3) $$R_{period,i}=\frac{∆N_{broke,i}}{N_{i-1}}\times 100\%$$

$∆N_{broke,i}$ is the number of newly ruptured microcapsules within the *i_i_-th* time interval, with *i* = 1,2,3,4,5,6.

$N_{i-1}$ is the total number of microcapsules at the beginning of the interval.

**Figure 9:**
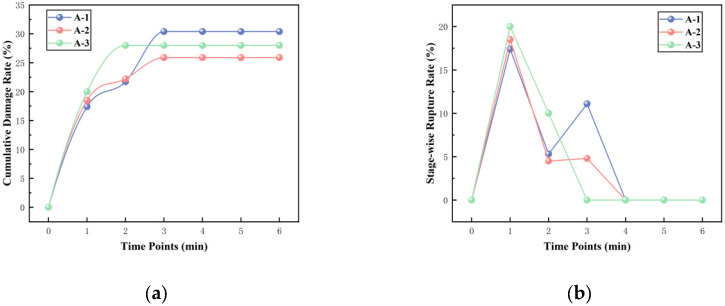
Rupture rate of microcapsules in an alkaline environment. (**a**) Cumulative rupture rate. (**b**) Interval rupture rate.

The results are shown in Table 11 and Figure 9.

The experimental results and calculations indicate that the rupture behavior of the microcapsule population exhibits distinct stages: rapid rupture followed by stabilization.

Stage 1: Rapid Rupture Phase. During this phase, the number of microcapsules decreased significantly, and the interval rupture rate rapidly climbed to a peak. In the three parallel experiments, the cumulative rupture rate at 15 min reached approximately 30%. Upon immersion in a high-alkali solution, a steep ion concentration gradient forms between the concentrated internal sodium silicate solution and the external medium. This gradient generates a substantial osmotic pressure difference across the shell. Driven by osmotic pressure, water molecules diffuse inward through nano-scale pores in the shell, increasing the internal pressure within the microcapsule. For microcapsules with inherent shell defects (e.g., localized thin spots, microcracks, regions of loose particle packing), this osmotic pressure is sufficient to cause rapid swelling and rupture.

Stage 2: Stabilization Phase. After approximately 15 min, the number of microcapsules stabilized, the interval rupture rate dropped to zero, and the cumulative rupture rate curve entered a plateau. From the perspective of film diffusion control theory, for microcapsules with structurally intact and dense shells, the physical barrier formed by the close-packed arrangement of nano-silica particles effectively hinders the inward diffusion of OH^−^ ions. The attack of OH^−^ on silica is a slow process controlled by interfacial reaction, limited by the diffusion rate of OH^−^ through the dense shell. Within the short time window of 30–45 min, the diffusion distance is limited, and the depth of erosion is insufficient to penetrate the shell and induce structural failure. Therefore, microcapsules with intact structures remain stable within this timescale.

**Figure 10:**
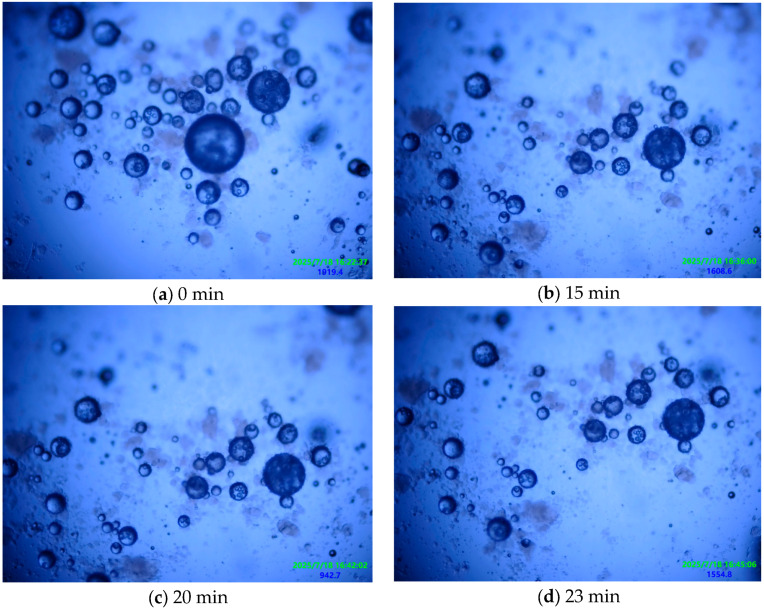

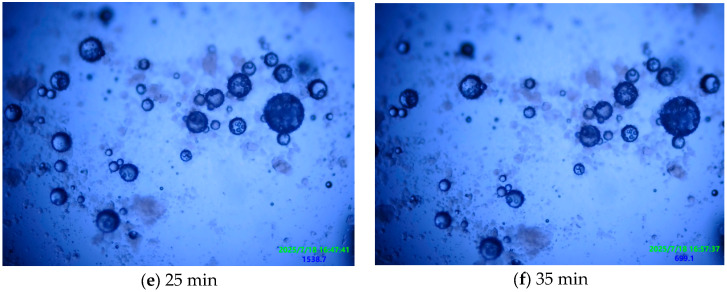
In situ fluorescence microscopy observation of microcapsule morphology and quantity in an alkaline environment (time series). All images were taken at ×400 magnification and include instrument-generated scale bars in the lower-left corner.

Figure 10 below shows the macroscopic observation results from one set of experiments.

When the temperature rose to 30 °C, the pH of all experimental groups showed a significant jump, marking the initiation of release behavior. This temperature threshold is highly consistent with the LCST range. When the temperature exceeds the LCST, hydrogen bonds between HPMC molecular chains and water molecules are disrupted. Hydrophobic interactions, dominated by groups like methyl and hydroxypropyl, lead to cooperative dehydration and coiling of the molecular chains. This microscopic phase transition translates into isotropic contractile internal stress within the confined microcapsule. When the accumulated stress exceeds the strength of the shell, it induces crack initiation and propagation. As temperature continued to rise, the microcapsules maintained release, with pH values in all groups increasing steadily, but the release rate slowed down. This indicates that microcapsule release is not instantaneous but rather a gradual process. From a release kinetics perspective, this sustained release mode can be attributed to the superposition of two factors

The alkali resistance of the microcapsules is mainly attributed to the following two reasons:

1. Hydrophobic Modification of the Shell Material: The hydrophobic nano-silica used underwent surface modification. This hydrophobic treatment extensively covers reactive sites like silanol groups (Si-OH) on the nanoparticle surface, significantly reducing their reactivity and thus slowing down the erosion reaction.
2. Dense Packing of the Shell Material: As observed in the microscopic morphologies (Figure 3 and Figure 7), the microcapsules prepared under the synergistic action of low temperature and shear force possess a shell where nano-silica particles are closely and orderly packed, forming a dense composite structure with low porosity. This structure limits the diffusion rate of OH^−^ ions within the shell and restricts the reaction rate, thereby greatly delaying the corrosion and penetration behavior caused by hydroxide ions and effectively retarding the damage rate of the shell material in the alkaline environment.

In summary, this study successfully constructed temperature-responsive trigger units. These units have complete structures and remain stable for a short time in the highly alkaline cement environment. This result provides key stability evidence for the application of microcapsules in cement-based materials.

## 5. Conclusions

Aiming to overcome the challenge of encapsulating highly alkaline sodium silicate, this paper proposed a microcapsule design concept based on a stimuli-responsive composite core system. Microcapsules with a hydrophobic nano-silica shell and a sodium silicate–HPMC composite core were successfully prepared using a low-temperature shear method. The influence of temperature on the preparation process, the temperature-triggered release properties of the microcapsules, and their short-term stability in an alkaline environment were systematically investigated. The main conclusions are as follows:

1. Low temperature is key to successful encapsulation. At room temperature, HPMC and sodium silicate undergo irreversible side reactions, preventing the system from forming microcapsules. Under low-temperature conditions, side reactions are inhibited, and the solution viscosity is adjusted to a suitable range. Mechanical shear can then effectively break it into stable small droplets, which are ultimately encapsulated by nano-silica. The optimal preparation window is 10–15 °C.
2. The microcapsules exhibit a distinct temperature-triggered release behavior. The phase transition characteristic of HPMC acts as the release switch. Upon heating, HPMC chains coil, generating internal stress that induces shell rupture. The release process shows temperature-dependent gradual characteristics. Drying enhances shell strength but does not alter the fundamental triggering mechanism.
3. The microcapsules possess good short-term stability in an alkaline environment. In a highly alkaline solution, the cumulative rupture rate within 30 min is approximately 30%, primarily attributed to osmotic rupture of defective microcapsules. Structurally intact microcapsules remain stable, owing to the effective barrier against OH^−^ provided by the dense shell of hydrophobically modified nano-silica.

This paper validates the feasibility of the composite core concept and successfully prepared a temperature-sensitive microcapsule. More importantly, it provides a simple, controllable process and design philosophy for microcapsule preparation. Future work can focus on shell toughening, particle size reduction, and investigating the long-term behavior of these microcapsules within cement matrices.

## Figures and Tables

**Table 1:** Orthogonal experimental design for temperature and HPMC dosage.

Experiment No.	Temperature (T, °C)	HPMC Dosage (C, wt.%)
1	5	0
2	5	1
3	5	2
4	5	4
5	5	8
6	15	0
7	15	1
8	15	2
9	15	4
10	15	8
11	25	0
12	25	1
13	25	2
14	25	4
15	25	8

**Table 2:** Parameters for the temperature gradient experiments in microcapsule preparation.

Preparation Temperature /°C	25	20	15	10	5
ID	T 1	T 2	T 3	T 4	T 5

**Table 3:** Microcapsule temperature sensitivity test.

ID	Microcapsules /g	H_2_O /g	Initial Water Temperature /°C	Dried or Not
D 1-1	0.5	200	25	No
D 1-2	1	400	25	No
D 1-3	1.5	600	25	No
D 2-1	0.5	200	25	Yes
D 2-2	1	400	25	Yes
D 2-3	1.5	600	25	Yes

**Table 4:** Experimental design for alkaline resistance test of microcapsules.

ID	Preparation Temperature	Sodium Silicate Solution pH	Observation Duration/min	Time Interval/min
A-1	20	13.2	30–40	5
A-2	15	13.2	30–40	5
A-3	10	13.2	30–40	5

**Table 6:** Calibration curve of sodium silicate concentration vs. pH at 25 °C.

Sodium silicate concentration	0	0.05	0.1	0.15	0.2	0.3	0.4
pH (25 °C)	7.5	7.86	8.63	9.24	9.57	10.58	11.7

**Table 7:** Cumulative release rate data of sodium silicate from microcapsules at 25–70 °C (duplicate tests). Fitting results of release kinetic models.

Temperature/°C	Measured Average pH (D1)	Measured Average pH (D2)	Ct (D1) g/L	Ct (D2) g/L	Qt (D1) %	Qt (D2) %
25	7.57	7.6	0.0070	0.0099	2.375%	3.359%
30	9.19	8.94	0.1609	0.1374	54.599%	46.624%
35	9.32	9.22333	0.1732	0.1644	58.772%	55.786%
40	9.46	9.43667	0.1866	0.1847	63.319%	62.674%
45	9.61	9.58667	0.2009	0.1990	68.171%	67.526%
50	9.80	9.76333	0.2189	0.2158	74.279%	73.227%
60	10.30	10.29	0.2664	0.2659	90.397%	90.227%
70	10.52	10.59333	0.2872	0.2947	97.455%	100%

**Table 8:** Fitting results of release kinetic models.

D1:		
Kinetic Model	Linear Fitting Equation	R^2^
Zero-order model	Q_t_ = 0.0781·t + 0.4479	0.8902
First-order model	ln(1 − Q_t_) = −0.1597·t − 0.3122	0.9415
Higuchi model	Q_t_ = 0.1791·t + 0.3517	0.9624
Ritger–Peppas model	lnQ_t_ = 0.7218·lnt − 0.4122	0.9931
D2:		
Kinetic Model	Linear Fitting Equation	R^2^
Zero-order model	Q_t_ = 0.0911·t + 0.3571	0.9301
First-order model	ln(1 − Q_t_) = −0.1701·t − 0.2295	0.9578
Higuchi model	Q_t_ = 0.2052·t + 0.2548	0.9786
Ritger–Peppas model	lnQ_t_ = 0.8105·lnt − 0.5803	0.9947

**Table 9:** EDS analysis results of the precipitates on ruptured microcapsules (area in Figure 6).

Element Number	Element Symbol	Element Name	Atomic Conc. /%	Weight Conc. /%
6	C	Carbon	14.26	9.90
8	O	Oxygen	61.11	56.50
11	Na	Sodium	21.67	28.80
14	Si	Silicon	2.96	4.80

**Table 10:** Number of microcapsules over time in an alkaline environment.

Time Point	Number of Microcapsules		
	A-1	A-2	A-3
0	23	27	25
1	19	22	20
2	18	21	18
3	16	20	18
4	16	20	18
5	16	20	18
6	16	20	18

**Table 11:** Cumulative and interval rupture rates of microcapsules in the alkaline environment.

Time Point	$R_{cum,i}$			$R_{period,i}$		
	A-1	A-2	A-3	A-1	A-2	A-3
0	0	0	0	0	0	0
1	17.4	18.5	20	17.4	18.5	20
2	21.7	22.2	28	5.3	4.5	10
3	30.4	25.9	28	11.1	4.8	0
4	30.4	25.9	28	0	0	0
5	30.4	25.9	28	0	0	0
6	30.4	25.9	28	0	0	0

## Data Availability

The original contributions presented in the study are included in the article, further inquiries can be directed to the corresponding author.
